# *CALR* but Not *JAK2* Mutations Are Associated with an Overexpression of Retinoid X Receptor Alpha in Essential Thrombocythemia

**DOI:** 10.3390/cancers16081511

**Published:** 2024-04-16

**Authors:** Ana Guijarro-Hernández, Cristina Hurtado, María José Larráyoz, María José Calasanz, José Luis Vizmanos

**Affiliations:** 1 Department of Biochemistry and Genetics, School of Sciences, University of Navarra, 31008 Pamplona, Spain; aguijarro@alumni.unav.es (A.G.-H.); churtado@unav.es (C.H.); 2 CIMA LAB Diagnostics, Department of Biochemistry and Genetics, School of Sciences, University of Navarra, 31009 Pamplona, Spain; mjlarra@unav.es (M.J.L.); mjcal@unav.es (M.J.C.)

**Keywords:** myeloproliferative neoplasms, ET, CALR, *RXRA*

## Abstract

**Simple Summary:**

Although the most well-known molecular mechanism in essential thrombocythemia (ET) is the activation of JAK2/STAT, the study of mechanisms that are independent of JAK2/STAT activation related to the disease is on the rise. Previous studies in a *C. elegans* model revealed that the overexpression of *nhr-2* counterparts could be a JAK2/STAT-independent mechanism, derived from *CALR* mutations in ET patients. In this work, we evaluated the expression of potential orthologs of *nhr-2* in human cell lines and in mononuclear cells from ET patients with *CALR* or *JAK2* mutations. The results show that mutant calreticulin is associated with an overexpression of *RXRA* in patients with ET, which could be relevant in the disease, pointing to the need for future research testing retinoids and other drugs targeting RXRα for the treatment of these patients.

**Abstract:**

Essential thrombocythemia (ET) is a blood cancer caused by mutations in *JAK2* and *CALR*. It is widely recognized that both mutations lead to the constitutive activation of JAK2/STAT signaling, although other JAK/STAT-independent pathogenic mechanisms triggered by these alterations have also been described in ET. In an attempt to study JAK2/STAT-independent mechanisms derived from *CALR* mutations, our research group created a *C. elegans* model with patient-like mutations in calreticulin that lacks JAK counterparts. The introduction of patient-like mutations in the calreticulin of *C. elegans* leads to an increase in the transcriptional expression of *nhr-2*, independently of JAK2/STAT activation. In the present study, we aim to verify if this mechanism is conserved in patients with ET harboring *CALR* mutations. To do so, we evaluated the expression of potential orthologs of *nhr-2* in human cell lines of interest for the study, as well as in bone marrow (BM) or peripheral blood (PB) mononuclear cells from patients with *CALR* or *JAK2* mutations. The results revealed that this mechanism is conserved in *CALR*-mutated ET patients, since *CALR*, but not *JAK2* mutations, were associated with an overexpression of *RXRA* in patients with ET. The use of drugs targeting the activation or blockade of this target in the analyzed cell lines did not result in changes in cell viability. However, *RXRA* might be relevant in the disease, pointing to the need for future research testing retinoids and other drugs targeting RXRα for the treatment of ET patients.

## 1. Introduction

Essential thrombocythemia (ET) is a rare blood disorder, characterized by increased platelet production and a greater risk of thrombotic events. Patients with ET are generally asymptomatic and have a virtually normal life expectancy, unless the disease progresses to myelofibrosis (MF). However, some patients may present symptoms such as splenomegaly or thrombohemorrhagic complications. To date, therapies have aimed to alleviate the symptoms presented by patients, but no drug therapy has been able to influence survival or cure the disease. As mutations in *JAK2* and *CALR* have been associated with the condition in most cases, many attempts have been made to study the molecular mechanisms triggered by these mutations in order to propose new therapeutic strategies. It is well known that mutations in these genes lead to the constitutive activation of JAK2/STAT signaling, although other JAK2/STAT-independent pathogenic mechanisms of these mutated proteins have also been described (reviewed in [1]).

In an attempt to study these mechanisms, our research group created a model in *C. elegans* with patient-like mutations in calreticulin. This model is suitable for studying JAK2/STAT-independent mechanisms of mutant calreticulin, as it does not have JAK counterparts. Thus, all the effects generated by the introduction of the mutated calreticulin in this model should be a consequence of mechanisms that are independent of JAK2/STAT. In previous studies, we observed that the introduction of patient-like mutations in the calreticulin of *C. elegans* led to an increase in the transcriptional expression of *nhr-2* (Nuclear Hormone Receptor 2), which seems to be involved in transcription regulation and cell differentiation [2]. Based on these findings, we searched for possible human orthologous genes of *nhr-2* to determine if an increase in their expression was also derived from mutant calreticulin in patients with ET, which would point to new potential therapeutic targets in these patients.

## 2. Materials and Methods

### 2.1. Cell Culture

SET-2, MARIMO, and K562 cells were cultured in 80% RPMI 1640 (Gibco™ RPMI-1640, Invitrogen-Life Technologies, Paisley, UK) and 20% fetal bovine serum (FBS, HyClone Fetal Bovine Serum, Thermo Fisher Scientific, Essex, UK) at 37 °C with 5% CO_2_. Additionally, the medium was supplemented with 1% penicillin/streptomycin (Gibco™ Penicillin-Streptomycin 10,000 U/mL, Invitrogen-Life Technologies, Paisley, UK) to prevent bacterial contamination.

### 2.2. Patient Samples

Patient samples used in this study were obtained with written informed consent from the patient and were completely anonymized. Mononuclear cells were extracted with Ficoll from the bone marrow (BM) or peripheral blood (PB) of 38 patients with ET and *CALR* mutations (26 with type 1 mutations and 12 with type 2 mutations), 21 patients with ET and the p.V617F JAK2 mutation, and 10 healthy donors as controls. Most of the samples from the patients were taken at diagnosis (Table S1).

### 2.3. qPCR

Total RNA was extracted using TRIzol^®^ (Thermo Fisher Scientific Inc., Paisley, UK) following the manufacturer’s specifications. The extracted RNA was purified with the commercial DNA-free^TM^ DNA Removal kit (Thermo Fisher Scientific Baltics UAB, Vilnius, Lithuania) and reverse-transcribed with Invitrogen™ M-MLV-RT and random hexamers. The expression of the genes of interest was analyzed by qPCR using primers and PrimeTime™ probes from IDT (Integrated DNA Technologies Inc., Coralville, IA, USA) (Table S2). All qPCR reactions were performed in 384-well plates using the CFX384™ Touch™ Real-Time PCR Detection System (Bio-Rad Laboratories, Hercules, CA, USA) with an amplification profile of 2 min at 50 °C, 10 min at 95 °C, and 40 cycles of 95 °C for 15 s, followed by 1 min at 60 °C. The amplification reactions were performed in a final volume of 10 µL per well with 5 µL of iTaq™ Universal Probes Supermix (Bio-Rad Laboratories, Hercules, CA, USA), 0.5 µL of PrimeTime™ primers and probes (20×) (IDT, Integrated DNA Technologies Inc., Coralville, IA, USA), 1 µL of cDNA (12.5 ng/µL), and 3.5 µL of nuclease-free water. Each sample was analyzed in triplicate, and appropriate negative controls were included. C_T_ values were collected using the CFX Manager™ software v3.1 (Bio-Rad Laboratories, Hercules, CA, USA). The expression level of each gene was normalized to the expression of *TBP* (#Hs.PT.58v.39858774 PrimeTime™ predesigned qPCR Assays; Integrated DNA Technologies Inc., Coralville, IA, USA).

### 2.4. Western Blot

Protein extraction was performed on 500,000 cells per cell line in triplicate. After washing with PBS (Gibco™, Thermo Fisher Scientific Inc., Paisley, UK) and discarding the supernatant, cells were lysed in 80 μL of RIPA Buffer (25 mM Tris-HCl at pH 7.6, 150 mM NaCl, 0.1% (*w/v*) sodium dodecyl sulfate (SDS), 1% (*w/v*) sodium deoxycholate, 1% (*v/v*) IGEPAL^®^ CA-630 (Sigma-Aldrich Co., St. Louis, MO, USA)), and protease inhibitors (cOmplete™, Roche^®^ Diagnostics GmbH, Mannheim, Germany). Samples were sonicated, and the protein concentration was quantified using the Pierce™ BCA Protein Assay Kit (Thermo Fisher Scientific Inc., Rockford, IL, USA) following the specifications from the manufacturer.

For Western Blot, proteins were first separated by SDS-PAGE on 12% denaturing acrylamide gels. For this, 30 μg of protein was diluted in 8 μL of 4× Laemmli loading buffer (Bio-Rad Laboratories, Hercules, CA, USA), and 10% of β-mercaptoethanol and distilled water were immediately added to the final volume of 35 μL. Samples were then heated at 100 °C for 5 min and loaded onto the gel. Electrophoresis was performed for 1.5 h at 120 V and room temperature. After that, proteins were transferred to 0.45 μm pore nitrocellulose membranes (Whatman^®^ Protran^®^, Merck-Millipore, Billerica, MA, USA) for 1 h at 350 mA and 4 °C in a Mini Trans-Blot^®^ Cell system (Bio-Rad Laboratories, Hercules, CA, USA) with a transfer buffer composed of 25 mM Tris, 192 mM glycine, and 20% methanol. Next, non-specific binding sites were blocked by incubating the membrane in 10–20 mL of 5% (*w/v*) skimmed milk or BSA dissolved in TBS (20 mM Tris and 150 mM NaCl) with 0.1% Tween at RT for at least 1 h with agitation. After that, the membranes were incubated at 4 °C overnight with shaking and 5 mL of a solution of primary antibodies, which were specific for the detection of each protein, diluted in the same solution prepared for blocking: anti-RXRα (#3085, Cell Signaling Technology^®^, Danvers, MA, USA), diluted 1:1000 in 5% milk; anti-PPARγ (#2435, Cell Signaling Technology^®^, Danvers, MA, USA), diluted 1:1000 in 5% BSA; and anti-β-actin (#8457, Cell Signaling Technology^®^, Danvers, MA, USA), diluted 1:10,000 in 5% BSA. The next day, the membranes were washed three times with TBS with 0.1% Tween for 10 min to remove excess primary antibody. Subsequently, they were incubated with shaking for 1 h at room temperature in 5 mL of secondary anti-rabbit IgG antibody linked to HRP (#7074, Cell Signaling Technology^®^, Danvers, MA, USA), diluted 1:2000 (for anti-RXRα and anti-PPARγ) or 1:10,000 (for anti-β-actin) in 5% BSA. The membranes were then additionally washed three more times with TBS with 0.1% Tween for 10 min to remove excess secondary antibody.

For detection, the commercial system Lumi-Light^PLUS^ Western Blotting Substrate (Roche Diagnostics GmbH, Mannheim, Germany) was used according to the instructions from the manufacturer. The chemiluminescent signal intensity was detected using a ChemiDoc XRS+ system coupled with the Bio-Rad Universal Hood II system (Bio-Rad Laboratories, Hercules, CA, USA) and quantified using ImageJ v1.53 software [3]. The expression levels of the target proteins were normalized by dividing the signal obtained for the protein in each sample by that obtained for β-actin in the same sample from the same gel. Membrane images were cropped to show results that were specific to the proteins of interest. All uncropped Western Blot images can be found in Figure S1.

### 2.5. Cell Line Treatments and Viability Assessment

Cell line treatments were carried out in 96-well ELISA plates, with a final volume of 100 μL per well (300 cells/μL). All compounds were dissolved in DMSO at concentrations that allowed for the exposure of the cells to the desired doses of compound, while adding less than 0.5% DMSO to avoid its cytotoxic effects. Controls consisted of wells containing cells without DMSO and with the maximum amount of DMSO added in each treatment. Each treatment and control were tested in triplicate on each of the cell lines. Plates were incubated for 72 h in a Galaxy B incubator (RS Biotech Laboratory Equipment Ltd., Irvine, UK) at 37 °C with 5% CO_2_.

To determine the number of viable cells after exposure to the different treatments, a colorimetric assay was performed using the commercial system CellTiter 96^®^ AQueous One Solution Cell Proliferation Assay (Promega Corp., Madison, WI, USA). To do this, 20 μL of the reagent was added to each well that already contained a volume of 100 μL. The plates were incubated for 2.5 h at 37 °C with 5% CO_2_. Subsequently, absorbance at 492 nm was measured using a Multiskan Ex Microplate Photometer (Thermo Fisher Scientific Inc., Waltham, MA, USA). It was verified that the added DMSO had no cytotoxic effects on the cells by comparing the viability obtained in the control of cells without compound with that of cells with the highest percentage of DMSO used. Finally, the percentage of live cells after exposure to each treatment was calculated, considering 100% viability for the wells to which DMSO without compound was added.

### 2.6. Statistics

Statistical calculations were performed using StataSE v12 software (StataCorp LP, College Station, TX, USA). The significance level (α) was set at 0.05. Differences were considered non-significant (ns) when *p* > 0.05, significant (*) when *p* < 0.05, very significant (**) when *p* < 0.01, and highly significant (***) when *p* < 0.001. All data obtained in this study were analyzed using an ANOVA test followed by multiple comparisons, except for the gene expression data of patients. In this case, the median test was used, followed by multiple comparisons, as the residuals did not fit a normal distribution or follow a similar distribution between groups for any of the genes analyzed. For the statistical analysis of qPCR, when no expression was detected in any of the samples, it was considered that the C_T_ value was 40, since it is the detection limit of the system (above this cycle, no signal is detected). In these cases, it is important to note that the differences are at least as significant as indicated in the graphs, but it cannot be ruled out that they may be higher than indicated (if the C_T_ value is greater than 40).

## 3. Results and Discussion

**Figure 1:**
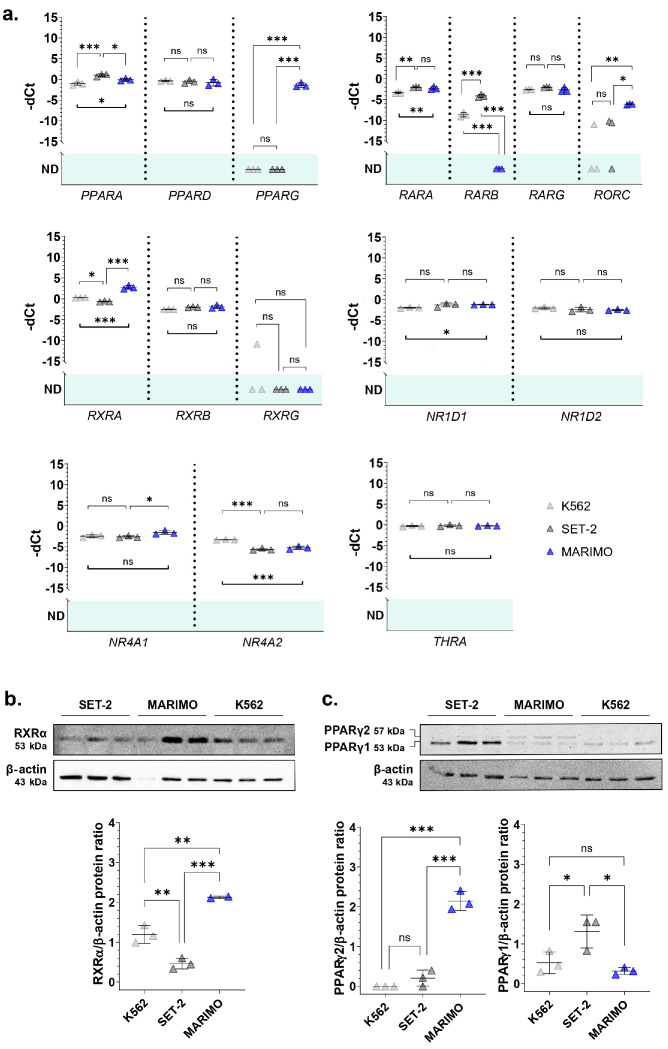
(**a**) Normalized expression of some of the *nhr-2* orthologs in the human cell lines K562, SET-2, and MARIMO. Individual values (*n* = 3), means, and standard deviations are shown. When no expression was detected in any of the samples analyzed, no statistic is shown (ND, not detected). (**b**,**c**) Analysis of RXRα and PPARγ expression by Western Blot in the cell lines K562, SET-2, and MARIMO. The antibody for PPARγ detects both isoforms PPARγ1 and PPARγ2. Individual values (*n* = 3), means, and standard deviations are shown in the graphs. Differences were considered non-significant (ns) when *p* > 0.05, significant (*) when *p* < 0.05, very significant (**) when *p* < 0.01, and highly significant (***) when *p* < 0.001.

When performing a BLASTP search of the NHR-2 protein sequence provided by UniProt (Q10902) using UniProtKB/Swiss-Prot reference proteomes for *Homo sapiens* [NCBI:txid9606] as the target database, many homologous sequences were found (Figure S2). From all of them, we selected the genes with a percentage of identity in the encoded protein sequence above 30% with NHR-2 that could be of interest for ET patients (previously related to hematological disorders) (Table S3) and analyzed their expression in cell lines derived from *CALR-* or *JAK2*-mutated patients with leukemic transformation of ET by qPCR. We focused on the cell lines SET-2 (with a heterozygous p.V617F mutation in *JAK2* [4] and established from an ET patient at megakaryoblastic leukemic transformation [5]) and MARIMO (with a heterozygous type 1 *CALR* mutation [6] and established from an ET patient with a therapy-related acute myeloid leukemia [7]). We also included in the analysis the cell line K562 (with the fusion *BCR::ABL1* established from a pleural effusion of a patient with chronic myeloid leukemia in terminal blastic crisis [8]) as a control of a myeloproliferative neoplasm other than from ET. As we hypothesized that the overexpression of *nhr-2* counterparts could be due to a JAK2/STAT-independent mechanism derived from mutant calreticulin, we expected that these orthologs would only be overexpressed in the MARIMO cell line (*CALR*-mutated) and not in the SET-2 cell line (*JAK2*-mutated) when compared to the control cell line K562. The results of the qPCR analyses showed that this pattern of expression was true for *RXRA*, *PPARG*, and *RORC* (Figure 1a). These three genes encode nuclear receptors that regulate the transcription of multiple target genes, yet their regulatory mechanisms and functions differ. Thus, RXRα (retinoid X receptor alpha) and PPARγ (peroxisome proliferator-activated receptor gamma) are activated by retinoids and fatty acids, respectively. In addition, RXRα can bind to its target genes by forming homodimers or heterodimers, while PPARγ generally binds DNA as heterodimers with RXRXα [9,10]. Interestingly, both genes have previously been found to be altered in myeloid leukemia cells and proposed as treatments for myeloid cancers [11,12,13]. Although the functions of RORC (nuclear receptor ROR-gamma) are not well understood, it is well known that this nuclear receptor has key functions in lymphoid organogenesis and thymopoiesis, but no function has been described in the myeloid tissue [14]. Therefore, we decided to focus our attention on a deeper analysis of *RXRA* and *PPARG*.

Then, we verified that the *RXRA* and *PPARG* expression data were correlated at the protein level using Western Blot. The results show that RXRα was also overexpressed in the MARIMO cell line (Figure 1b). Regarding PPARγ, differences were found in the isoform PPARγ2, which is also only overexpressed in the MARIMO cell line (Figure 1c).

Given these results, using qPCR, we analyzed the expression of *RXRA* and *PPARG* in mononuclear cells extracted with Ficoll from the BM or PB of 38 patients with ET and CALR mutations (26 with type 1 mutations and 12 with type 2 mutations), 21 patients with ET and the p.V617F *JAK2* mutation, and 10 healthy donors as controls.

**Figure 2:**
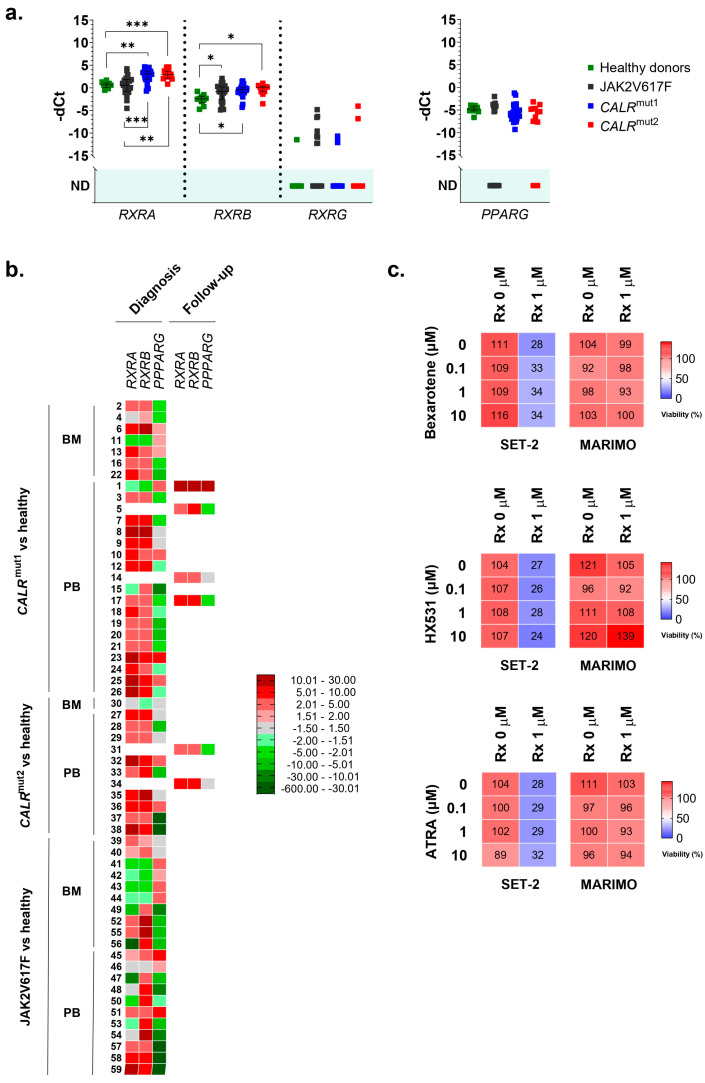
(**a**) Normalized expression of human *nhr-2* orthologs *RXRA*, *RXRB*, *RXRG*, and *PPARG* in samples from 38 patients with ET and mutations in *CALR* (26 with type 1 mutations and 12 with type 2 mutations), 21 patients with ET and mutations in *JAK2*, and 10 healthy donors. Individual values, means, and standard deviations are shown. When no expression was detected in any of the samples analyzed, no statistic is shown (ND, not detected). (**b**) Individual heatmaps with *RXRA*, *RXRB*, *RXRG*, and *PPARG* expression data in the samples. Some of the samples were extracted from bone marrow (BM) and others from peripheral blood (PB). Additionally, the time of sample collection is indicated, either at the time of diagnosis or during follow-up of the disease. In all cases, average fold change values are represented from shades of green (negative fold change) to shades of red (positive fold change). (**c**) The percentage of viable cells quantified by the MTS assay after exposing MARIMO and SET-2 cell lines to increasing concentrations (0, 0.1, 1, and 10 μM) of bexarotene, HX531, and ATRA without ruxolitinib or in combination with ruxolitinib at 1 μM (*n* = 3). * *p* < 0.05, ** *p* < 0.01, and *** *p* < 0.001.

In the series of analyzed samples, no differences were found for *PPARG*. On the contrary, *RXRA* was highly overexpressed in *CALR*-mutated patients, but not in *JAK2*-mutated patients, compared to healthy controls (Figure 2a,b). This overexpression was observed in 23 of 26 patients with type 1 *CALR* mutations (88.5%) and in 11 of 12 patients with type 2 *CALR* mutations (91.7%). In contrast, only 9 of the 21 patients with the *JAK2* mutation (42.8%) had *RXRA* overexpressed compared to healthy donors. No significant differences were found in the *RXRA* expression between samples taken from BM and those from PB. Additionally, all samples taken during the disease follow-up from patients with *CALR* mutations (6/6) were overexpressing *RXRA*, regardless of the type of mutation. This result is consistent with the results obtained in cell lines and corroborates that the overexpression of *RXRA* could be triggered by mutant type 1 and type 2 calreticulins in a *JAK2*-independent manner.

As *RXRA* was found to be overexpressed in these patients, we decided to study the expression of other members of the RXR family (*RXRB* and *RXRG*). Regarding *RXRB*, we found it to be overexpressed in samples from patients with the *JAK2* mutation (16 out of 21 patients analyzed, 76.2%) and also in samples from patients with *CALR* mutations (36 out of 38 patients analyzed, 94.7%) (Figure 2a,b). Therefore, this expression cannot be considered an event that is independent of the activation of the JAK2 axis. In this case, no different expression patterns were observed depending on the origin of the sample (BM or PB) or the moment of their collection (at diagnosis or during disease follow-up) (Figure 2b). Finally, *RXRG* expression was undetectable in the majority of samples, and no significant differences were found between healthy donors and patients with ET, regardless of whether they had *CALR* or *JAK2* mutations (Figure 2a).

Thus, the results point to the fact that the overexpression of *RXRA* seems to be a consequence of mutant calreticulin in humans. For this reason, we tried to test an RXR-targeted treatment in SET-2 and MARIMO cell lines, expecting to find some delay in proliferation in the MARIMO cell line due to *RXRA* overexpression. Specifically, bexarotene (#SML0282, Sigma-Adrich Co., St. Louis, MO, USA), HX531 (#SML2170, Sigma-Adrich Co., St. Louis, MO, USA), and ATRA (#R2625, Sigma-Adrich Co., St. Louis, MO, USA) were tested as, respectively, a pan-RXR agonist, a pan-RXR antagonist, and a compound derived from vitamin A that acts as a ligand for retinoic acid receptors and X retinoid receptors. Each of these compounds were also tested in combination with ruxolitinib (#HY-50856, MedChemExpress MCE^®^, Monmouth Junction, NJ, USA), a JAK1/JAK2 inhibitor that is approved for patients with MF post-ET, to find possible synergies and interactions. It is important to note that MARIMO cells are resistant to ruxolitinib [6]. Our results show that bexarotene, HX531, and ATRA had no effects at the tested concentrations on the viability of the *CALR*-mutated MARIMO cell line, either alone or in combination with ruxolitinib (Figure 2c). As expected, a decrease in viability was observed in the SET-2 cell line after exposure to 1 μM ruxolitinib [15], but the combination of this compound with increasing doses of bexarotene, HX531, and ATRA also did not change the effects of ruxolitinib (Figure 2c).

These results led us to consider whether the *CALR*-mutated MARIMO cell line was indeed a suitable model for evaluating the action of these compounds. It should be noted that this cell line shows other alterations in addition to a *CALR* mutation [16], which could dampen the effects produced by compounds targeting RXRs. In fact, although it shows an *MPL* mutation (p.S505N), it does not show the canonical activation of the JAK2/STAT5 cascade that is present in patients with mutations in *CALR*, probably due to these cells not expressing MPL. The replicative potential of MARIMO cells seems to be more related to the activation of the MAPK pathway by a p.Q61K mutation in *NRAS* [6,16,17].

Despite this, the magnitude of *RXRA* overexpression that was observed in almost all the samples analyzed in patients with ET and *CALR* mutations and recent studies that highlight the importance of RXRα and retinoids in myeloid malignancies [12,18,19,20] point to a possible relevant effect on the disease. Among the results of these studies, it is worth noting that an RXRα/RXRβ deficiency has been shown to induce the exhaustion of HSCs and the differentiation of myeloid cells, causing a myeloproliferative-type disease in mice [18]. Therefore, the overexpression of *RXRA* in *CALR*-mutated ET patients could be a protective mechanism against the progression of the disease, which might be potentiated pharmacologically. For this reason, it does not seem appropriate to completely discard the RXR-targeted treatments for ET, either alone or in combination with ruxolitinib, although further analyses in a more suitable model are necessary.

## 4. Conclusions

In conclusion, this study shows that type 1 and type 2 mutant calreticulins could lead to the overexpression of RXRA in patients with ET and points to the need for future research testing retinoids and other drugs targeting RXRα for the treatment of these patients.

## Data Availability

All available data can be accessed by contacting the corresponding author.

## Supplementary Materials

The following supporting information can be downloaded at https://www.mdpi.com/article/10.3390/cancers16081511/s1: Figure S1: Uncropped Western Blot images; Figure S2: Results of a BLASTP search of the NHR-2 protein sequence provided by UniProt (Q10902) using UniProtKB/Swiss-Prot reference proteomes for Homo sapiens [9606] as the target database; Table S1: Samples from patients with ET and healthy donors included in the gene expression study; Table S2: Primers and probes used for qPCR analysis; Table S3: NHR-2 human orthologs (*C. elegans*) selected for subsequent analyses based on the BLASTP results (more than 30% identity) and a previous association with hematological diseases.

## References

[1] Guijarro-Hernández A., Vizmanos J.L. (2021). A broad overview of signaling in *Ph*-negative classic myeloproliferative neoplasms. Cancers.

[2] Guijarro-Hernández A., Eder-Azanza L., Hurtado C., Navarro-Herrera D., Ezcurra B., Novo F.J., Cabello J., Vizmanos J.L. (2023). Transcriptomic analysis reveals JAK2/MPL-independent effects of calreticulin mutations in a *C. elegans* model. Cells.

[3] Schneider C.A., Rasband W.S., Eliceiri K.W. (2012). NIH Image to ImageJ: 25 years of image analysis. Nat. Methods.

[4] Quentmeier H., MacLeod R.A., Zaborski M., Drexler H.G. (2006). JAK2 V617F tyrosine kinase mutation in cell lines derived from myeloproliferative disorders. Leukemia.

[5] Uozumi K., Otsuka M., Ohno N., Moriyama T., Suzuki S., Shimotakahara S., Matsumura I., Hanada S., Arima T. (2000). Establishment and characterization of a new human megakaryoblastic cell line (SET-2) that spontaneously matures to megakaryocytes and produces platelet-like particles. Leukemia.

[6] Kollmann K., Nangalia J., Warsch W., Quentmeier H., Bench A., Boyd E., Scott M., Drexler H.G., Green A.R. (2015). MARIMO cells harbor a *CALR* mutation but are not dependent on JAK2/STAT5 signaling. Leukemia.

[7] Yoshida H., Kondo M., Ichihashi T., Hashimoto N., Inazawa J., Ohno R., Naoe T. (1998). A novel myeloid cell line, Marimo, derived from therapy-related acute myeloid leukemia during treatment of essential thrombocythemia: Consistent chromosomal abnormalities and temporary *C-MYC* gene amplification. Cancer Genet. Cytogenet..

[8] Lozzio C.B., Lozzio B.B. (1975). Human chronic myelogenous leukemia cell-line with positive Philadelphia chromosome. Blood.

[9] Keller H., Givel F., Perroud M., Wahli W. (1995). Signaling cross-talk between peroxisome proliferator-activated receptor/retinoid X receptor and estrogen receptor through estrogen response elements. Mol. Endocrinol..

[10] Okuno M., Arimoto E., Ikenobu Y., Nishihara T., Imagawa M. (2001). Dual DNA-binding specificity of peroxisome-proliferator-activated receptor gamma controlled by heterodimer formation with retinoid X receptor alpha. Biochem. J..

[11] Welch J.S., Niu H., Uy G.L., Westervelt P., Abboud C.N., Vij R., Stockerl-Goldstein K.E., Jacoby M., Pusic I., Schroeder M.A. (2014). A phase I dose escalation study of oral bexarotene in combination with intravenous decitabine in patients with AML. Am. J. Hematol..

[12] Di Martino O., Ferris M.A., Hadwiger G., Sarkar S., Vu A., Menéndez-Gutiérrez M.P., Ricote M., Welch J.S. (2022). RXRA DT448/9PP generates a dominant active variant capable of inducing maturation in acute myeloid leukemia cells. Haematologica.

[13] Esmaeili S., Salari S., Kaveh V., Ghaffari S.H., Bashash D. (2021). Alteration of *PPAR-GAMMA* (*PPARG*; *PPARγ*) and *PTEN* gene expression in acute myeloid leukemia patients and the promising anticancer effects of *PPARγ* stimulation using pioglitazone on AML cells. Mol. Genet. Genom. Med..

[14] Kurebayashi S., Ueda E., Sakaue M., Patel D.D., Medvedev A., Zhang F., Jetten A.M. (2000). Retinoid-related orphan receptor gamma (RORgamma) is essential for lymphoid organogenesis and controls apoptosis during thymopoiesis. Proc. Natl. Acad. Sci. USA.

[15] Szymańska J., Smolewski P., Majchrzak A., Cebula-Obrzut B., Chojnowski K., Treliński J. (2015). Pro-apoptotic activity of ruxolitinib alone and in combination with hydroxyurea, busulphan, and PI3K/mTOR Inhibitors in *JAK2*-positive human cell lines. Adv. Clin. Exp. Med..

[16] Han L., Czech J., Maurer A., Brümmendorf T.H., Chatain N., Koschmieder S. (2018). Mutant *NRAS* Q61K is responsible for MAPK pathway activation in the MARIMO cell line and renders these cells independent of the CALR-MPL-JAK2-STAT5 pathway. Leukemia.

[17] Wang C., Hu X., Wan Y., Wang S., Qi K., Li Y., Qiao J., Zeng L., Li Z., Fu C. (2021). The synergistic inhibitory effect of combining MK-2206 and AZD 6244 in MARIMO cells harboring a calreticulin gene mutation. Chemotherapy.

[18] Menéndez-Gutiérrez M.P., Porcuna J., Nayak R., Paredes A., Niu H., Núñez V., Paranjpe A., Gómez M.J., Bhattacharjee A., Schnell D.J. (2023). Retinoid X receptor promotes hematopoietic stem cell fitness and quiescence and preserves hematopoietic homeostasis. Blood.

[19] Rajamani B.M., Illangeswaran R.S.S., Benjamin E.S.B., Balakrishnan B., Jebanesan D.Z.P., Das S., Pai A.A., Vidhyadharan R.T., Mohan A., Karathedath S. (2023). Modulating retinoid-X-receptor alpha (RXRA) expression sensitizes chronic myeloid leukemia cells to imatinib in vitro and reduces disease burden in vivo. Front. Pharmacol..

[20] Qiu F., De Thé H. (2021). An exciting RXRA mutant revives interest in retinoids for acute myeloid leukemia. Haematologica.

