# Supplementary material for: CALR but Not JAK2 Mutations Are Associated with an Overexpression of Retinoid X Receptor Alpha in Essential Thrombocythemia
Source: Cancers (Basel). 2024 Apr 16;16(8):1511. doi: 10.3390/cancers16081511 (PMC11048154; doi:10.3390/cancers16081511)
Supplement: Supplementary file 1 [file cancers-16-01511-s001.zip › Figures S1 and S2.pdf]

Supplementary Materials

CALR but not *JAK2* mutations are associated with an overexpression of Retinoid X Receptor Alpha in essential thrombocythemia

Ana Guijarro-Hernández [1], Cristina Hurtado [1], María José Larráyoz [2], María José Calasanz [2] and José Luis Vizmanos [1,*]

[1] Department of Biochemistry and Genetics, School of Sciences, University of Navarra, 31008 Pamplona, Spain
[2] CIMA LAB Diagnostics, Department of Biochemistry and Genetics, School of Sciences, University of Navarra, 31009 Pamplona, Spain
* Correspondence: jlvizmanos@unav.es

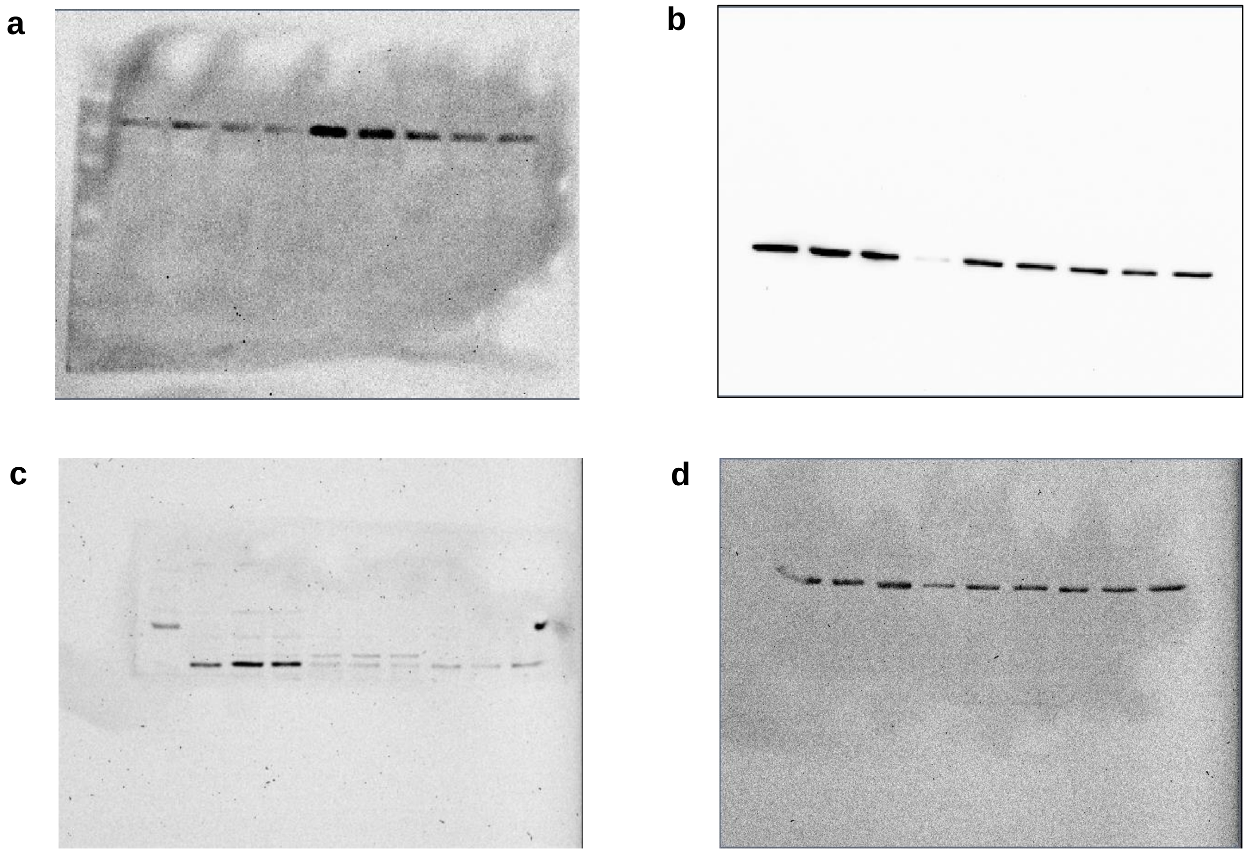

Figure S1. Uncropped Western Blot images. **(a)** RXRα. **(b)** B-actin for RXRα samples. **(c)** PPARγ. **(d)** B-actin for PPARγ samples.

Please find the full Figure S2 on the following page.

Figure S2. Results of a BLASTP search of the NHR-2 protein sequence provided by UniProt (Q10902) using UniProtKB/Swiss-Prot reference proteomes for Homo sapiens [9606] as the target database.

blastp (version: BLASTP 2.12.0+)
Database: uniprotkb_refprotswissprot
Sequence: EMBOSS_001
Length: 472

Tue, May 02, 2023 at 10:49:41
Tue, May 02, 2023 at 10:53:05

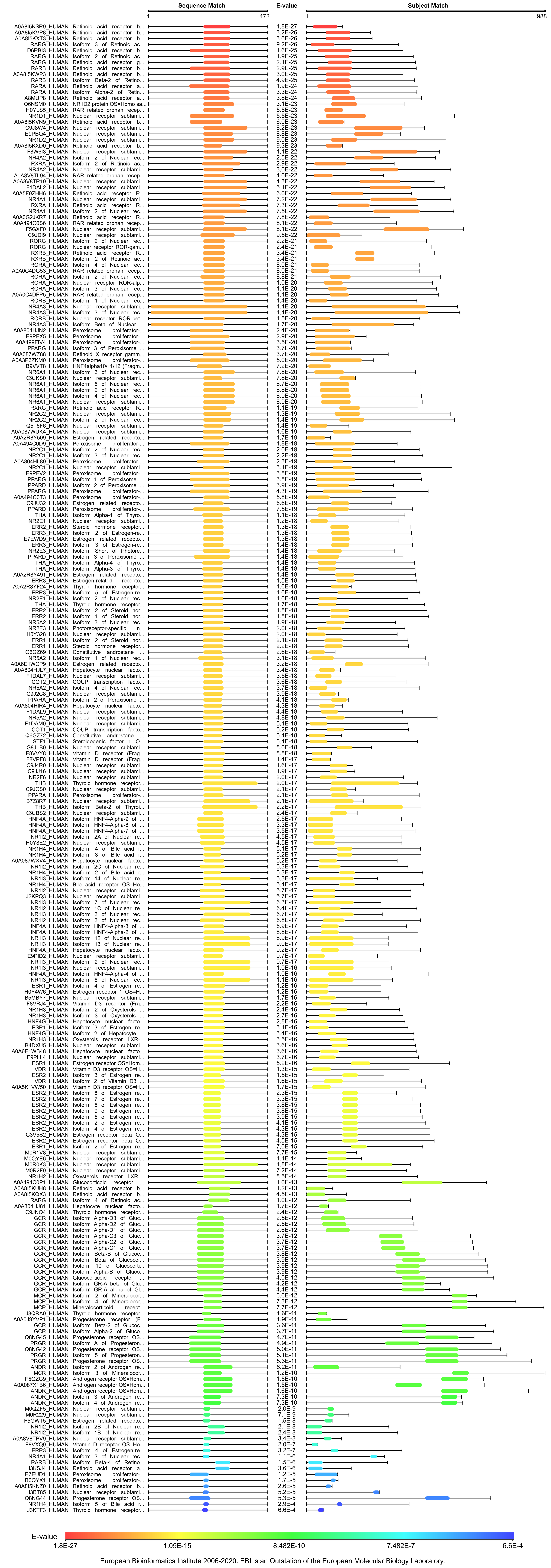

European Bioinformatics Institute 2006-2020. EBI is an Outstation of the European Molecular Biology Laboratory.
